# Visualization of flow dynamics in the portal circulation using 320-detector-row computed tomography: a feasibility study

**DOI:** 10.1186/s41747-020-00197-8

**Published:** 2021-01-12

**Authors:** Ken Kageyama, Akira Yamamoto, Atsushi Jogo, Shinichiro Izuta, Daisuke Himoto, Akihiko Kakimi, Etsuji Sohgawa, Yukio Miki

**Affiliations:** 1 Department of Diagnostic and Interventional Radiology, Graduate School of Medicine, Osaka City University, 1-4-3 Asahi-machi, Abeno-ku, Osaka, 545-8585 Japan; 2 Department of Central Radiology, Osaka City University Hospital, 1-5-7 Asahi-machi, Abeno-ku, Osaka, 545-8586 Japan

**Keywords:** Four-dimensional computed tomography, Hypertension (portal), Multidetector computed tomography, Portal vein, Portasystemic shunt

## Abstract

Multidetector row computed tomography (CT) scanners perform dynamic scanning and have a wide scan range. Time-resolved three-dimensional CT (*i.e.*, 4D CT) has recently enabled visualization of flow in neurovascular vessels. We hypothesized that 4D CT technology would be a useful and non-invasive method for visualizing the flow dynamics of the portal circulation. The aim of this study was to evaluate the technical feasibility of 4D CT for visualizing flow dynamics in the portal circulation using 320-detector-row CT. 4D CT images of 18 consecutive patients with portal circulation including gastrorenal shunt were retrospectively evaluated for their ability to generate flow dynamics of the portal circulation. Flow dynamics could be visualized by 4D CT in 68 of the 72 vessels in the portal vein, splenic vein, superior mesenteric vein, and gastrorenal shunt. Flow direction could not be identified in four vessels, all of them being superior mesenteric veins. Flow direction was recognized on 4D CT in the 68 vessels of the portal circulation. A preliminary validation study revealed that flow direction of all 19 vessels in the portal circulation had concordance between 4D CT and color Doppler ultrasound. 4D CT could visualize flow dynamics of the portal circulation.

**Supplementary Information:**

The online version contains supplementary material available at 10.1186/s41747-020-00197-8.

## Key points

- Four-dimensional computed tomography (4D CT) could visualize flow dynamics in the portal circulation.
- Radiologists could recognize flow direction from the flow dynamics of the portal circulation on 4D CT.
- Flow direction in the portal circulation demonstrated concordance between 4D CT and color Doppler ultrasound.

## Background

Portal hypertension leads to serious complications, including bleeding from gastroesophageal varices, ascites, and portosystemic encephalopathy, in patients with hepatic cirrhosis. Hepatic venous pressure gradient is the gold standard method for the diagnosis of portal hypertension [1]; however, as it is measured by catheter, it is highly invasive. Endoscopic evaluation of varices is not only an indirect method of assessing portal hypertension, but it is also an invasive technique.

Noninvasive techniques proposed in recent decades for the measurement of portal hypertension include flow imaging using ultrasound (US) or magnetic resonance imaging (MRI) [1–6]. Doppler US and two-dimensional phase-contrast MRI are widely performed and well validated for measuring velocity and flow direction [7–9]. Doppler US is a simple technique used to evaluate portal hemodynamics without radiation exposure; however, this technique has relatively poor reproducibility and accuracy characterized by intraobserver and interobserver variation, and factors such as gas-filled bowels, obesity, and complex anatomy hinder observation of vessels in the portal circulation [3, 4, 10–12]. Although two-dimensional phase-contrast MRI can overcome these obstacles, elaborate preparation is necessary to define the plane in which all portal vessel flows are measured. Four-dimensional (4D) flow MRI is a recently developed technique that generates three-dimensional visualization of vessels, time-resolved flow images, and quantification of hemodynamics [6, 13]. Although several 4D flow studies have been conducted, multicenter validation has not yet been performed [13]. A disadvantage of 4D flow MRI imaging is the longer scan time required to obtain flow data and anatomical data compared with Doppler US.

Although conventional computed tomography (CT) technology cannot produce time-resolved flow images of the portal circulation in an efficient manner, CT studies are generally performed in patients with portal-hypertension-related diseases to determine the portal anatomy prior to interventional radiology (IR) procedures such as balloon-occluded retrograde transvenous obliteration, transjugular intrahepatic portosystemic shunt, and percutaneous transhepatic sclerotherapy. Despite the radiation burden to the patient, CT has significant advantages over US and MRI in terms of planning operative procedures, understanding the complicated anatomy of the portosystemic shunt, determining whether or not to catheterize, and deciding which vein from multiple portosystemic shunts should be embolized prior to therapy [14]. CT is also superior to US and MRI for detecting the tiny multiple drainage veins connected to the portosystemic shunt [15]. After IR treatment, CT is the modality of choice for evaluating thrombosis of varices or portosystemic shunts after embolization [16, 17].

Regarding portal-hypertension-related diseases, IR treatments raise serious post-therapeutic concerns that include the emergence of ascites, deterioration of other varices, portal thrombosis, and liver failure [16, 17]. Although each modality has drawbacks, recent US and 4D flow MRI studies have revealed the relationship between portal flow and the deterioration of gastroenteric varices and liver function [2, 18–20]. IR treatments can alter the flow dynamics of the portal circulation between pre- and post-therapy, resulting in changed liver function [18, 20]. If flow dynamic imaging could be performed by 4D flow CT in addition to routine CT scans, it would simplify the preoperative examination for patients and would indicate portosystemic shunts that should and should not be embolized. The addition of 4D flow CT images of the portal circulation to routine CT images could potentially improve assessment of the liver and could also be used to evaluate treatment efficacy.

Multidetector row CT scanners perform dynamic scanning and have a wide scan range. Time-resolved three-dimensional CT (*i.e.*, 4D CT) has recently enabled visualization of flow in neurovascular vessels [21]. We hypothesized that 4D CT would be a useful and non-invasive method for visualizing the flow dynamics of the portal circulation. The aim of this study was to evaluate the technical feasibility of 4D CT with 320-detector row CT for visualizing flow dynamics in the portal circulation.

## Methods

This retrospective study was approved by our Institutional Committee on Human Investigation. Informed consent for 4D CT in addition to routine CT imaging was obtained from all patients prior to 4D CT scanning. From March 2018 to September 2019, 66 patients underwent IR treatment for such as balloon-occluded retrograde transvenous obliteration and percutaneous transhepatic sclerotherapy at our department. After excluding 27 patients with diagnoses other than gastrorenal shunt, 39 patients with gastric varices and/or portosystemic encephalopathy including gastrorenal shunt met the criteria for 4D CT prior to IR treatment. Of these, the following were also excluded: 10 patients who were scanned less than 3 months prior to IR treatment, 6 who had a history of varix rupture, and 5 with renal failure. A final total of 18 consecutive patients (9 men, 9 women; mean age 65.3 years; age range 41–83 years; mean weight 61.7 kg, weight range 46–83 kg) who had undergone 4D CT before IR procedures were enrolled in the study.

### 4D CT protocol

4D CT was acquired by a 320-detector-row CT scanner (Aquilion one, Canon Medical Systems, Otawara, Japan) equipped with 320 × 0.5-mm-wide detector rows covering a 16-cm-long volume per rotation. A bolus of contrast medium was injected by an automatic injector via the cubital vein, followed by a saline flush. To visualize the aorta at the level of the celiac artery bifurcation, a bolus-tracking scan was performed after intravenous injection of 10 mL of contrast medium (Oypalomin 300, Fuji Pharma, Tokyo, Japan) into the cubital vein to confirm enhancement of the aorta and celiac artery. After the bolus-tracking scan, 600 mgI/kg of contrast medium was injected at a rate of 3.7–4.8 mL/s over 20 s, followed by 20 mL of saline, during 4D CT scanning. When contrast enhancement of the aorta at the level of the celiac artery bifurcation reached > 100 HU, patients were asked to inhale and hold their breath and 4D CT scanning was initiated. Scan parameters were as follows: detector collimation 320 × 0.5 mm; matrix 512 × 512; slice thickness 0.5 mm; tube voltage 120 kVp; mean tube current 58.8 mA (range, 40–80 mA); gantry rotation time 0.5 s; interval 1 s; number of imaging volumes 18; total scan time 26 s. Temporal resolution was 1.0 s, corresponding to the interval time. The current was determined based on twice the CT dose index for commonly performed helical CT examinations such as portal phase imaging. In this protocol, a series of 18 dynamic volumes was obtained, consisting of 320 slices covering the portal circulation. To make it easier for patients to hold their breath during scanning, they were given oxygen via a mask beforehand to increase their oxygen saturation [22].

After 4D CT scanning, portal, venous, and late phase imaging was performed from the diaphragm to the pelvis. Portal and venous phase acquisitions were initiated 35 and 55 s after the start of 4D scanning. The late phase scan was initiated 180 s after injection of contrast medium. Scan parameters were as follows: detector collimation 320 × 0.5 mm; matrix 512 × 512; table feed 65.04 mm/s; slice thickness 0.5 mm; tube voltage 120 kVp; mean tube current 392.8 mA (range, 277.5–562.5 mA); gantry rotation time 0.5 s; interval 0.5 mm.

### Post-processing of 4D CT images

The 18 phases of the 4D CT images were transferred to a workstation (PhyZiodynamics; Ziosoft, Tokyo, Japan) for post-processing. This commercially available software creates smooth video images using a motion coherence function that fills in interphase motion with interpolated data. Using this software, we generated two additional phases between the original phases to create interphase motion, thus creating partially artificially constructed smooth and clear 4D motion [23]. To display only the portal circulation, the abdominal arteries were removed from the cine images, and only portal vessels with enhancement > 70 HU were displayed. Finally, a total of 52 phases were reconstructed into three-dimensional volume-rendering and maximum intensity projection images. Multiple-frame dynamic cine loops from the anterior and inferior aspects were generated for evaluation. A radiographer generated dynamic cine loops of the maximum intensity projection and volume-rendering images of all patients in multiple views, which took 3–5 h to create, depending on the experience of the radiographer.

### Evaluation of 4D CT images

Two radiologists, each of them with more than 15 years of experience in diagnostic and interventional radiology, independently evaluated all 4D CT examinations. A 2-point scale (visualized or not visualized) was used to assess visualization of flow in the portal vein, splenic vein, superior mesenteric vein, and gastrorenal shunt. They watched videos of the flow dynamics in the portal vein, splenic vein, and superior mesenteric vein and assessed flow direction of these vessels using a 2-point scale (hepatopetal flow, toward the liver or hepatofugal flow, away from the liver) by consensus.

### Radiation doses of CT scans

Radiation doses were recorded in all 18 cases for the 4D CT scans and all other scans.

### Validation study with US

In the first 7 patients, color Doppler US was performed as a preliminary examination to assess the direction of hepatopetal or hepatofugal flow in the portal, splenic, and superior mesenteric veins, using a LOGIQ S7 Expert machine (GE Healthcare, Tokyo, Japan) equipped with a C1-5-D convex probe. US was performed with the patient in the supine position and was scheduled on the same day as 4D CT.

### Statistical analysis

No statistical analysis was performed due to complete concordance observed in the validation study.

## Results

**Fig 1:**
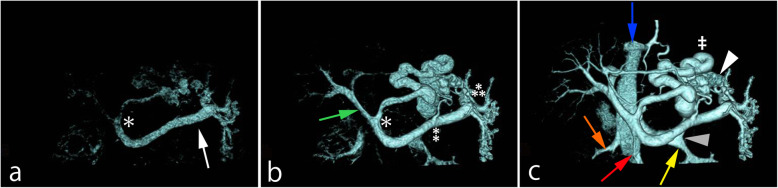
A 67-year-old female with gastric varices and hepatic encephalopathy. Volume rendering images, anterior aspect. **a** Early image depicts the splenic vein (white arrow) and left gastric vein (asterisk). **b** Mid-phase image additionally depicts the portal vein (green arrow), retro gastric vein (double asterisk), and short gastric vein (triple asterisk). **c** Late image depicts gastric varices (white arrowhead) and gastrorenal shunt (gray arrowhead) behind the splenic vein. The red, blue, yellow, and orange arrows indicate the superior mesenteric vein, inferior vena cava, left renal vein, and right renal vein, respectively. The portosystemic shunt (double dagger) is composed of afferent veins (left gastric, retro gastric, and short gastric veins) and an efferent vein (gastrorenal shunt)

**Fig. 2:**
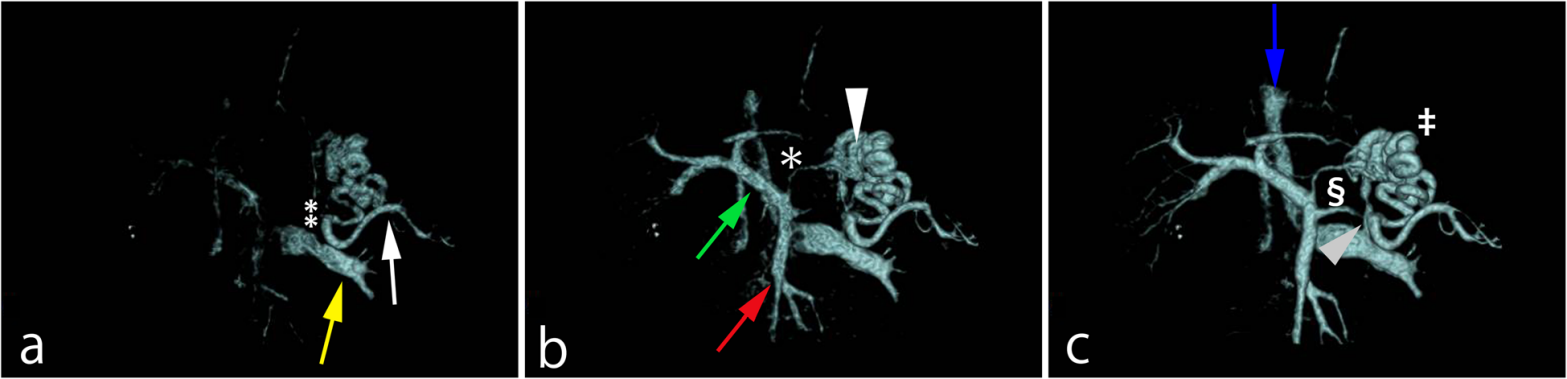
A 66-year-old male with gastric varices. Volume rendering images, anterior aspect. All of the portal venous system except the central splenic vein shows hepatopetal flow. **a** Early image depicts a peripheral splenic vein (white arrow), retro gastric vein (double asterisk), and the left renal vein (yellow arrow). **b** Mid-phase image additionally depicts the superior mesenteric and portal veins (red and green arrows, respectively), the left gastric vein (asterisk), and gastric varices (white arrowhead). **c** Late image depicts the gastrorenal shunt (gray arrowhead) behind the splenic vein, the inferior vena cava (blue arrow), and central splenic vein (section), which shows hepatofugal flow into the posterior gastric vein. The portosystemic shunt (double dagger) is composed of afferent veins (left gastric and retro gastric veins) and an efferent vein (gastrorenal shunt)

Flow dynamics could be visualized by 4D CT in 68 of the 72 vessels (portal vein, splenic vein, superior mesenteric vein, and gastrorenal shunt) in the 18 patients. Flow direction could not be identified by 4D CT in four superior mesenteric veins. The following scans performed after 4D CT visualized superior mesenteric veins in the 4 cases. In the 68 vessels that could be visualized by 4D CT, both readers recognized flow direction from the flow dynamics of the portal circulation on 4D CT. All patients had hepatopetal flow in the portal vein (Figs. 1 and 2, Supplemental data 1, 2, 3, 4). The splenic vein had hepatofugal flow in 6 patients (Fig. 2, Supplemental data 3 and 4, Table 1) and the superior mesenteric vein had hepatofugal flow in 1 patient (Table 1).

**Table 1:** Summary of patient characteristics

Patient characteristic					4D CT scan parameter					Portal phase parameter			Venous phase parameter			Late phase parameter			Total			Flow direction on CT				Flow direction on US		
Case number	Sex	Age (years)	Height (cm)	Weight (kg)	Voltage (kV)	Current (mA)	CTDI (mGy)	DLP(mGy·cm)	Effective dose(mSv)	CTDI (mGy)	DLP(mGy·cm)	Effective dose(mSv)	CTDI (mGy)	DLP(mGy·cm)	Effective dose(mSv)	CTDI (mGy)	DLP(mGy·cm)	Effective dose(mSv)	CTDI (mGy)	DLP(mGy·cm)	Effective dose(mSv)	Portal vein	Splenic vein	SMV	Gastrorenal shunt	Portal vein	Splenic vein	SMV
1	F	80	151	58	120	70	33.8	540.0	8.1	15.1	787.9	11.8	15.1	787.9	11.8	15.9	709.7	10.6	79.9	2,825.5	42.3	Pe	Fu	Pe	Fu	Pe	Fu	Pe
2	F	47	150	83	120	80	38.5	615.6	9.2	19.9	1,079.6	16.1	20.1	716.9	10.7	20.1	716.9	10.7	98.6	3,129	46.7	Pe	Fu	Pe	Fu	Pe	Fu	N
3	M	64	172	72	120	70	33.8	540.0	8.1	15.6	971	14.5	15.3	524.3	7.8	16.4	560.6	8.4	81.1	2,595.9	38.8	Pe	Pe	N	Fu	Pe	Pe	Fu
4	M	65	161	64	120	60	28.9	462.6	6.9	15.4	820.3	12.3	14.6	527.6	7.9	15.4	820.3	12.3	74.3	2,630.8	39.4	Pe	Pe	Pe	Fu	Pe	Pe	Pe
5	F	69	151	70	120	70	33.8	540.0	8.1	17	1,056.5	15.8	17.7	700.8	10.5	17.1	979.5	14.6	85.6	3,276.8	49	Pe	Pe	Fu	Fu	Pe	Pe	Fu
6	F	87	144	51	120	50	24.1	385.2	5.7	10.1	522.5	7.8	10.1	351.8	5.2	10.8	478.3	7.1	55.1	1,737.8	25.8	Pe	Pe	Pe	Fu	Pe	Pe	Pe
7	M	63	183	61	120	50	24.1	385.2	5.7	12.3	823.2	12.3	12.9	537.3	8.0	12.8	775.6	11.6	62.1	2,521.3	37.6	Pe	Fu	Pe	Fu	Pe	Fu	Pe
8	F	61	151	57	120	70	33.8	540.0	8.1	15.6	837.9	12.5	15.6	564.4	8.4	15.8	769.8	11.5	80.8	2,712.1	40.5	Pe	Fu	Pe	Fu	NA	NA	NA
9	F	41	149	71	120	70	33.8	540.0	8.1	16.8	1,030.1	15.4	17.3	710.5	10.6	16.9	965.9	14.4	84.8	3,246.5	48.5	Pe	Pe	N	Fu	NA	NA	NA
10	M	78	161	53	120	50	24.1	385.2	5.7	11	655.0	9.8	10.7	429.3	6.4	11.7	569.2	8.5	57.5	2,038.7	30.4	Pe	Pe	N	Fu	NA	NA	NA
11	F	83	155	46	120	40	19.2	307.8	4.6	10.5	568.1	8.5	10.2	375.2	5.6	11.2	544.8	8.1	51.1	1,795.9	26.8	Pe	Fu	Pe	Fu	NA	NA	NA
12	M	56	173	61	120	60	28.9	462.6	6.9	14.5	943.8	14.1	16.1	662	9.9	14.6	1,094.3	16.4	74.1	3,162.7	47.3	Pe	Pe	Pe	Fu	NA	NA	NA
13	M	66	151	50	120	40	19.2	307.1	4.6	10.6	611.8	9.1	9.9	373.8	5.6	10.9	547.7	8.2	50.6	1,840.4	27.5	Pe	Fu	Pe	Fu	NA	NA	NA
14	M	58	177	67	120	60	28.9	462.6	6.9	13	901.2	13.5	12.6	545	8.1	13.1	1,092.1	16.3	67.6	3,000.9	44.8	Pe	Pe	N	Fu	NA	NA	NA
15	M	67	172	68	120	60	28.9	462.6	6.9	14.4	1,147.9	17.2	14.4	1,147.9	17.2	15.6	858.7	12.8	73.3	3,617.1	54.1	Pe	Pe	Pe	Fu	NA	NA	NA
16	M	55	163	65	120	60	28.9	462.6	6.9	14.5	878.8	13.1	14.2	536.4	8.0	15.2	795	11.9	72.8	2,672.8	39.9	Pe	Pe	Pe	Fu	NA	NA	NA
17	F	69	159	57	120	50	24.1	385.2	5.7	12.2	882.1	13.2	12	522.7	7.8	13.6	730	10.9	61.9	2,520	37.6	Pe	Pe	Pe	Fu	NA	NA	NA
18	F	67	167	56	120	60	28.9	462.6	6.9	11	937.8	14.0	11	1,039.1	15.5	11.5	884.6	13.2	62.4	3,324.1	49.6	Pe	Pe	Pe	Fu	NA	NA	NA

*4D CT* Four-dimensional computed tomography, *CT* Computed tomography, *CTDI* CT dose index, *DLP* Dose-length product, *F* Female, *Fu* Fepatofugal flow away from the liver, *M* Male, *N* Not depicted, *NA* Not applicable, *Pe* Hepatopetal flow into the liver, *SMV* Superior mesenteric vein, *US* Ultrasound


**Additional file 1: Supplementary video 1.** A 67-year-old female with gastric varices and hepatic encephalopathy, anterior view. The video corresponds to Fig. 1.


**Additional file 2: Supplementary video 2.** A 67-year-old female with gastric varices and hepatic encephalopathy, caudal view. The video corresponds to Fig. 1 and Supplemental video 1. The caudal view enables separation of the gastrorenal shunt from the splenic vein.


**Additional file 3: Supplementary video 3.** A 66-year-old male with gastric varices, anterior view. The video corresponds to Fig. 2.


**Additional file 4: Supplementary video 4.** A 66-year-old male with gastric varices, caudal view. The video corresponds to Fig. 2 and Supplemental video 3. The caudal view enables separation of the gastrorenal shunt from the splenic vein.

The mean CT dose index, dose-length product, and effective dose of all 4D CT scans were 28.6 mGy (range 19.2–38.5), 458.2 mGy × cm (range 307.1–615.6), and 6.9 mSv (range 4.6–9.2), respectively (Table 1). The doses for the entire CT examination are reported in Table 1.

In the preliminary validation study, color Doppler US detected 20/21 vessels in the first seven patients, and 4D CT in these patients also detected 20/21 vessels (one vessel was not depicted on 4D CT in one patient, and one vessel was not depicted on US in one patient) (Table 1). After excluding these two vessels, there was a concordance of flow direction between 4D CT and US in 19/19 vessels.

## Discussion

This is the first study to visualize flow dynamics in the portal circulation on 4D CT with 320-detector-row computed tomography. As CT technology has evolved, the number of detectors has continued to increase, from 4-row CT scanners to the 320-detector-row CT scanners in current use. The most striking feature of multidetector CT is its broad scanning length in the z-axis. The 160-mm detector width enables acquisition of the portal circulation in a single rotation of the gantry and without couch movement. The established 4D flow MRI technique has a wider scan range and higher spatial resolution compared with our 4D-flow CT, in which the scan range is limited to 16 cm [13]. However, the advantages of the 4D CT method include short scan time, the ability to visualize the portal circulation at the same time as regular diagnostic imaging for a liver lesion or a gastric varix, and safety for patients with contraindication to MRI. The advantages of color Doppler US include the small size and portability of the US unit, non-invasiveness, and the short examination time with no radiation exposure. However, detection of portal vessels is dependent on operator skill and is made more difficult in the case of bowel gas and obesity [3, 4]. In the seven patients in the present preliminary study, one splenic vein could not be depicted on US due to obesity. The present results demonstrated that 4D CT might play a role as an additional imaging tool for understanding the portal circulation.

Breath-holding during 4D CT has important effects in terms of the patient’s oxygenation, portal flow dynamics, and detection of the portal circulation, but requires a high degree of patient cooperation. A previous report that compared the durations of prolonged breath-holding between preoxygenation and breathing room air [22] found that preoxygenation avoids hypoxemia and makes it easier for patients to hold their breath. Another study found that breath-holding after inspiration decreased portal blood flow [24]. In our study, we speculated that the Valsalva effect would lead to decreased portal flow during 4D CT scanning compared with breath-holding after expiration. As the breath-hold for 4D CT scanning should be as short as possible to reduce patient discomfort, we aimed for a scan time of less than 30 s. This is the reason why superior mesenteric venous flow was not detected in four of the present patients. The time difference in the arrival of the contrast medium happened between visualization of superior mesenteric venous flow and splenic venous flow. We prioritized visualization of splenic venous flow in order to visualize the short, retro, and left gastric veins as afferent feeders to a gastrorenal shunt. In addition, when the scan timing is adjusted to enable visualization of contrasted superior mesenteric venous flow, splenic venous flow cannot be seen because the splenic vein is already contrasted.

Previous studies have revealed changes in blood flow between pre-IR and post-IR treatment for gastrointestinal varices and hepatic encephalopathy using US and MRI [18, 20], increasing our understanding of changes in liver function due to changes in portal flow following IR therapies. In most patients of our study, the portal circulation had normal hepatopetal flow into the liver, except some patients with reversed hepatofugal flow in the splenic veins. These findings of flow direction are similar to the results of an US study of patients with gastric varices and hepatic encephalopathy [20]. US studies have also demonstrated that hepatofugal flow indicates the severity of the condition of the liver [2, 20]. Our preliminarily validation study revealed concordance of flow direction between 4D CT and US. By indicating flow direction, 4D CT may also predict the condition of the liver, although 4D CT provides limited detail regarding quantification of flow velocity and flow volume in the portal circulation compared with 4D MRI and US. Therefore, 4D flow CT imaging has potential as a diagnostic tool that could predict liver condition, emergence of complications, and therapeutic effects prior to commencement of IR treatment that can cause changes to dynamic flow in the portal circulation.

Compared with US and MRI, 4D CT is an emerging imaging method. As yet, the minimum radiation dose for 4D CT scanning is unknown, and therefore we are unable to compare the radiation exposure in our study and similar studies in terms of minimizing exposure. In this regard, we are able only to compare 4D CT scanning with conventional multiphasic CT as a reference. Effective radiation dose of 4D CT was lower than those of typical dynamic enhanced helical CT scans in this study. Helical scans usually covered whole abdominal cavity, whereas 4D CT scans covered only 16 cm. The radiation dose per unit length is higher for 4D CT than for typical helical CT [25]. However, the mean dose–length product in our series was 458.2 mGy × cm, which is similar to those reported previously for typical helical imaging of the upper abdomen with contrast medium (400–740 mGy × cm) [26, 27]. In further work, we will refine the examination protocol to keep radiation exposure as low as possible.

This study has some limitations. The first drawback is the limited breath-hold. Theoretically, it is possible to visualize superior mesenteric venous flow after visualization of splenic venous flow, but only if the patient can maintain a long enough breath-hold during 4D CT scanning. The second drawback is the small number of cases. The third drawback is lack of a control group consisting of healthy persons with normal portal circulation. The fourth drawback is that we did not evaluate 4D CT image quality, as there is no established imaging standard. Therefore, further investigations are required to enhance the validity of our results.

In conclusion, 4D CT could visualize flow dynamics in the portal circulation and might be a useful technique for capturing flow direction of the portal circulation.

## Data Availability

The relevant data have been included in the manuscript. The datasets used and analyzed during the current study are available from the corresponding author on reasonable request**.**

## Abbreviations

4D: Four-dimensional
CT: Computed tomography
IR: Interventional radiology
MRI: Magnetic resonance imaging
US: Ultrasound
